# Supplementary material for: The metalloproteinase inhibitor Marimastat improves skeletal muscle regeneration when administered intravenously after myonecrosis induced by the venom of Bothrops asper
Source: bioRxiv. 2026 Mar 27:2026.03.25.714270. Preprint. [Version 1] doi: 10.64898/2026.03.25.714270 (PMC13042068; doi:10.64898/2026.03.25.714270)
Supplement: Supplement 1 [file NIHPP2026.03.25.714270v1-supplement-1.pdf]

bioRxiv preprint doi: https://doi.org/10.64898/2026.03.25.714270; this version posted March 27, 2026. The copyright holder for this preprint (which was not certified by peer review) is the author/funder, who has granted bioRxiv a license to display the preprint in perpetuity. It is made available under aCC-BY 4.0 International license.

Supplementary files

SF-1

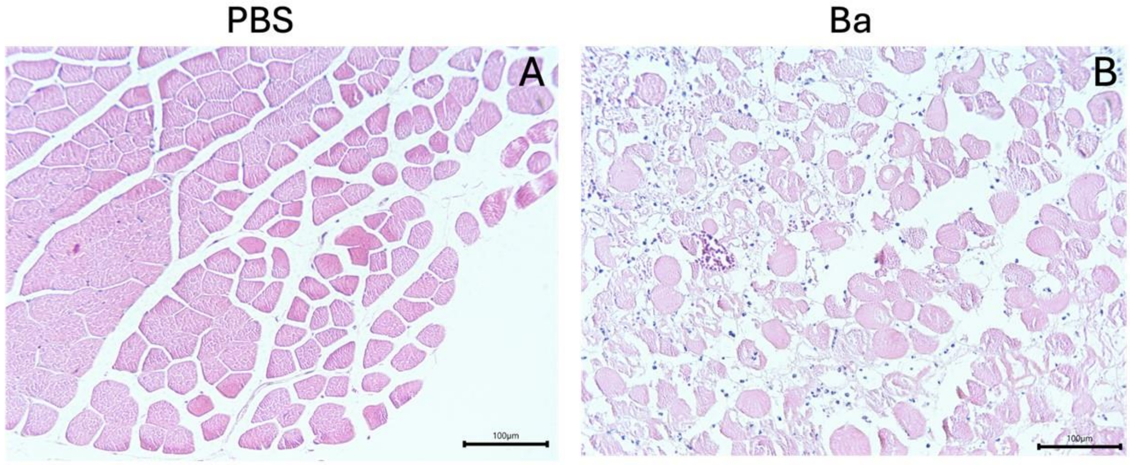

Supplementary figure S1. Light micrographs of sections of gastrocnemius muscles of mice receiving an i.m. injection of either PBS (A) of 50 µg *B. asper* venom (Ba), dissolved in 50 µL PBS. Tissue samples were obtained 24 h after injection and processed for embedding in paraffin and staining with hematoxylin-eosin. Notice the normal histological pattern in A and the prominent necrosis of muscle fibers in B. Bars represent 100 µm.

bioRxiv preprint doi: https://doi.org/10.64898/2026.03.25.714270; this version posted March 27, 2026. The copyright holder for this preprint (which was not certified by peer review) is the author/funder, who has granted bioRxiv a license to display the preprint in perpetuity. It is made available under aCC-BY 4.0 International license.

SF-2

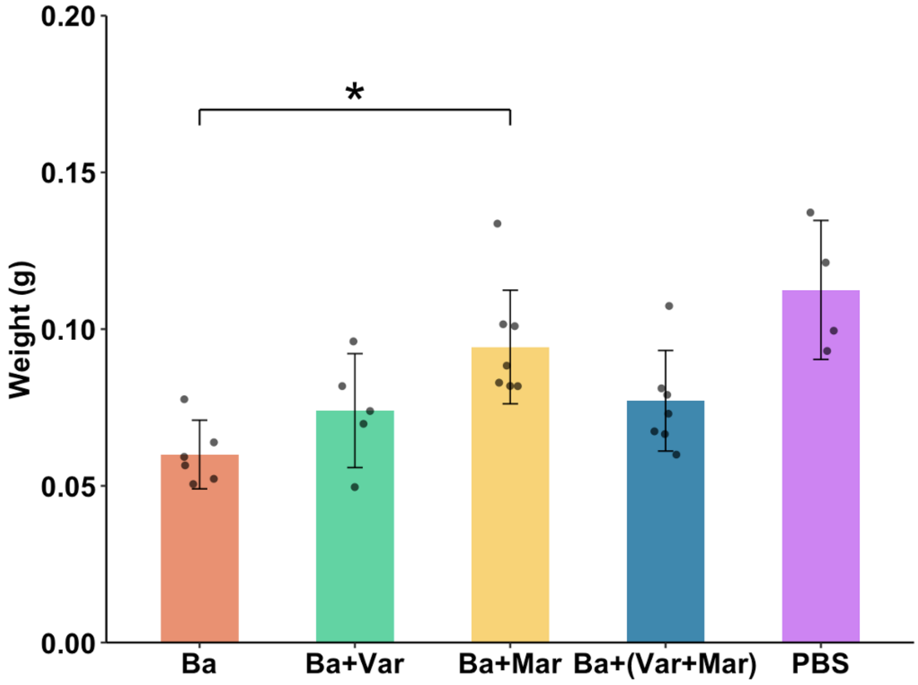

Supplementary figure S2. Wet weight (g) of the right gastrocnemius muscle of CD1 mice, at 28 days after intramuscular injection of 50 µg *B. asper* venom, dissolved in 50 µL PBS, followed, 24 h later, by the intravenous administration of either PBS (Ba), Varespladib (Ba + Var), Marimastat (Ba + Mar) or Varespladib + Marimastat (Ba + Var + Mar). The bar of PBS corresponds to non-envenomed mice receiving PBS. Wet weight of envenomed mice receiving Marimastat was significantly higher than that of envenomed mice not receiving inhibitors (*$p < 0.05$). Statistical analysis was performed using one-way ANOVA with Tukey *post-hoc* test. The weight of the muscle of mice injected with PBS only was significantly different from: Ba ($p < 0.001$), Ba+Var ($p < 0.05$) and Ba+(Var+Mar) ($p < 0.05$). No significant difference was observed between the treatments Ba+Mar and PBS ($p = 0.44$).
